# Effect of PreAnaesThesia Computerized Health (PATCH) Assessment on Duration of Nurse—Patient Consultation and Patient Experience: A Pilot Trial

**DOI:** 10.3390/ijerph17144972

**Published:** 2020-07-10

**Authors:** Tarig Osman, Eileen Lew, Elaine Lum, Jennifer Chew, Rajive Dabas, Ban Leong Sng, Josip Car

**Affiliations:** 1 Centre for Population Health Sciences, Lee Kong Chian School of Medicine, Nanyang Technological University, Singapore 308232, Singapore; tarigosm001@e.ntu.edu.sg (T.O.); elaine_lum@duke-nus.edu.sg (E.L.); josip.car@ntu.edu.sg (J.C.); 2 Department of Women’s Anaesthesia, KK Women’s and Children’s Hospital, Singapore 229899, Singapore; jennifer.chew.yl@kkh.com.sg (J.C.); rajive.dabas@singhealth.com.sg (R.D.); sng.ban.leong@singhealth.com.sg (B.L.S.); 3 Health Services and Systems Research, Duke-NUS Medical School Singapore 169857, Singapore; 4 Global eHealth Unit, Department of Primary Care and Public Health, School of Public Health, Imperial College London, London SW7 2AZ, UK

**Keywords:** preanaesthesia assessment, questionnaire, digital health

## Abstract

Preanaesthesia health assessment is gradually transitioning from paper-based, face-to-face assessment to digitized assessment, self-administered by the patient. This transition could potentially optimize the various goals of assessment, notably facilitating the efficient collection of the patient’s health information. We have previously developed and validated a tablet application (PreAnaesThesia Computerized Health assessment application or “PATCH”) for patients to conduct preanaesthesia self-assessment. In a randomized controlled trial, we sought to compare the duration of nurse–patient consultation and patient satisfaction between patients who underwent PATCH self-assessment vs. standard care nurse-led assessment. Fifty-two elective surgical patients were randomized to complete either PATCH assessment or standard care nurse-led assessment at an outpatient preoperative clinic. The duration of nurse–patient consultation was subsequently noted for all patients who also completed a satisfaction survey. The mean (SD) nurse–patient consultation times in the PATCH and standard care groups were comparable, at 11.5 (3.6) min and 12.2 (2.9) min, respectively (*p* = 0.703). Overall satisfaction scores were also comparable, at 23.9 and 27.0 respectively (*p* = 0.451) for the PATCH and standard nurse assessment groups. Favorable perceptions of PATCH among users ranged between 41.7% and 79.2%. In conclusion, PATCH self-assessment can feasibly be introduced into current practice with comparable nurse–patient consultation times and patient satisfaction.

## 1. Introduction

Globally, an estimated 313 million surgical procedures are performed each year [1]. Medical conditions among the surgical patient population are becoming increasingly complex, with a growing number of patients at the extremes of age [2,3]. Current anaesthesia practice mandates that patients undergo preanaesthesia health assessment (PHA) before the date of surgery. It is the first step in providing safe anaesthetic care by assessing the patient’s underlying co-morbid status, optimizing health and formulating patient-centric perioperative care plans [4,5,6,7]. This clinical assessment also aims to reduce perioperative morbidity and mortality, improve the cost effectiveness of perioperative care, reduce surgical delays and cancellations and return the patient to desirable functioning as quickly as possible [8].

PHA is often guided by a questionnaire which helps to identify at-risk patients and provide a record of medical history relevant to anaesthesia [9]. Specifically, prior studies have demonstrated the efficacy of standardized questionnaires in facilitating the assimilation of comprehensive patient information, the identification of patients at higher perioperative risks and the referral of patients to other specialists for further consultation [10,11]. The utilization of a questionnaire was also reported to be associated with a more efficient use of healthcare resources [12]. Conversely, PHA conducted without a standardized questionnaire could risk missing significant coexistent diseases which would then lead to patient harm, surgical delays or cancellation, indiscriminate or increased preoperative testing and increased healthcare costs [10,12].

With advancements in information technology, there has been a gradual shift from paper-based to digital versions of questionnaires used for PHA. Previous studies indicated that digital PHA was associated with accurate data collection, favorable patient perceptions and the accurate prediction of the American Society of Anesthesiologists (ASA) physical classification [13,14,15,16,17,18,19]. Hence, the development of digital PHA is perceived as a logical step in healthcare innovation—to elicit and enhance the assimilation of reliable and relevant historical information efficiently [18,20]. Digital PHA can also confer logistical benefits and improve the cost effectiveness of perioperative care [13,14,16].

The present study aims to evaluate the impact of digital PHA on nurse–patient consultation times at a preoperative clinic, compared with standard care nurse-led PHA conducted via an interview. The primary outcome was chosen based on the need to ensure clinic efficiency and throughput after the implementation of a new mode of screening. As secondary aims, we also evaluated patients’ perceptions of PreAnaesThesia Computerized Health (or PATCH) assessment and general satisfaction with the PHA process.

## 2. Materials and Methods

### 2.1. Study Context

At our hospital, PHA is conducted by a group of three trained triage nurses using a standardized paper questionnaire, administered via a face-to-face interview for all patients scheduled for same-day admission (SDA) surgery. In the same consultation, the triage nurse also shares relevant peri-operative information and instructions for the forthcoming surgery, manages the paperwork and answers queries. Based on pre-determined referral criteria, patients may be referred for further outpatient assessment with an anaesthetist after this initial PHA. Patients who do not meet the criteria are allowed to bypass outpatient assessment and turn up on the day of surgery to be assessed by their attending anaesthetists. Data from an internal audit (unpublished data) indicates that, on average, 900 elective surgical procedures are performed monthly, of which 50% are same-day admission (SDA) surgeries. This figure is predicted to increase with the growing patient preferences for ambulatory surgery. Thus, an opportunity exists to explore digitized PHA, to empower the patient in conducting preanaesthesia health self-assessments and optimize healthcare resources.

### 2.2. Study Design and Setting

This was a two-arm randomized equivalence pilot trial with a one-to-one subject ratio, conducted at the Preadmission Service (PAS) of KK Women’s and Children’s Hospital (KKH) from 6 June to 1 July 2019. KKH is the leading tertiary referral centre for obstetrics, gynaecology, neonatology and pediatrics in Singapore [21]. It is an 830-bed hospital with more than 500 specialists, accredited by the Accreditation Council for Graduate Medical Education International (ACGME-I) for the provision of specialist training and clinical research. The PAS is a one-stop clinic that facilitates the preoperative preparation of adult female patients (pediatric surgical patients are managed separately). Ethical approval was granted by the SingHealth Centralized Institutional Review Board (CIRB Ref:2017/3002) and the Nanyang Technological University Institutional Review Board (Ref:IRB-2017-12-011). The trial is registered with www.clinicaltrials.gov with the identifier NCT03737396.

### 2.3. Study Population

We recruited patients who were scheduled for SDA surgery. Inclusion criteria included: aged 21 years and above, proficient in spoken and written English, capable of using a tablet device and listed for SDA surgery. Patients who were listed for urgent operations, had a visual impairment or had completed the pro forma paper for PHA in the last month were excluded.

### 2.4. Sample Size and Randomization

**Figure 1:**
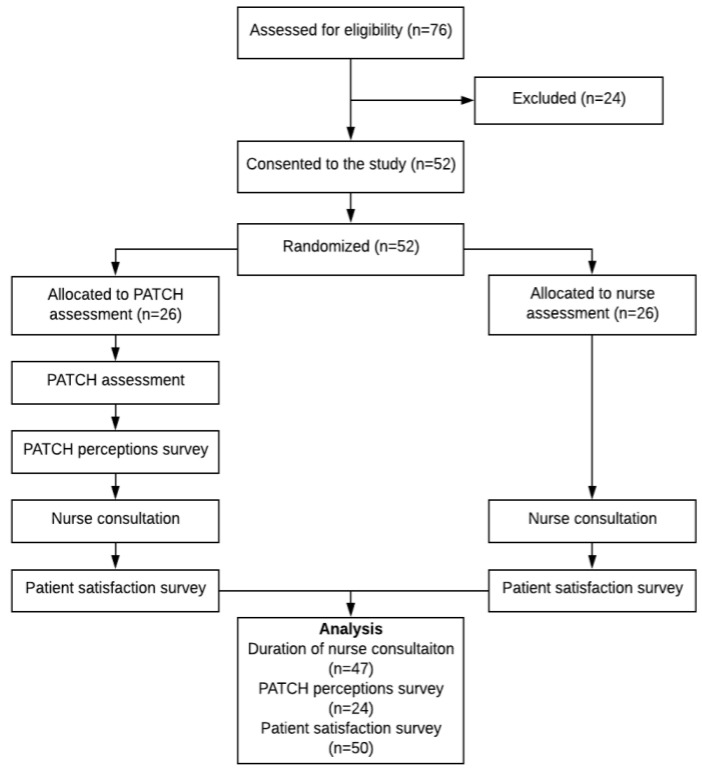
Flow chart detailing patient recruitment, randomization and allocation of patients to PreAnaesThesia Computerized Health (PATCH) and nurse assessments.

The study was powered for the primary outcome of the duration of nurse–patient consultation. Hospital statistics from December 2017 to May 2019 indicated that nurse–patient consultation times ranged from 11 to 14 min per patient. The sample size calculation was based on comparing two means. Using an expected difference in the mean time of three minutes, a standard deviation of 3.5 min at 80% power and a level of significance of 5%, the calculated sample size was 44. We aimed to recruit 52 patients to allow for a 2 × 2 block randomization, generated by Microsoft Excel version 16.0 (Office 365, Microsoft Corporation, Redmond, WA, USA) and allocation status was concealed using sealed envelopes. A study member who did not participate in the data collection generated the block randomization. The envelopes were arranged in the same order of randomization. The recruited patient was asked to draw an envelope that she then gave to the second research coordinator who informed her of her allocation status. The enrolment process, intervention allocation and analysis flowchart are shown in Figure 1.

### 2.5. Interventions

PATCH assessment consists of forty-one questions, of which thirty-eight have response options of “Yes, “No” and “Unsure”. A “Yes” response prompts the patient to provide more details in a free-text field. One question is presented on the screen at any one time. All data entered into PATCH were transferred in real-time via a secure WiFi connection to a server in the clinic.

Consenting patients randomized to undergo PATCH assessment were given instructions by the second research coordinator on how to operate and navigate the application on the tablet. Upon logging into the application using their unique queue numbers, patients also received information on the relevance of PATCH assessment. Patients subsequently completed the assessment un-aided and at their own pace while waiting for the nurse consultation. Before submission, the application would present a summary page for patients to check and edit their responses for a final time. After submission, patients had to complete a survey on their perceptions of PATCH, consisting of eight questions, with responses graded on a five-point Likert scale (5–Strongly agree, 4–Agree, 3–Neutral, 2–Disagree and 1–Strongly disagree). Free-text suggestions for improving PATCH were also solicited. Upon completion of the survey, patients proceeded with the nurse–patient consultation. Using the patient’s queue number, the PAS nurse accessed the patient’s data on a desktop version of PATCH (nurse’s interface) and reviewed the responses together with the patient, making amendments if necessary. The changes made by the nurse were documented in the audit log. Finally, the digital questionnaire was printed and signed by the patient before being filed into the health records, as mandated by hospital policy.

Patients randomized to the control arm underwent standard care and received PHA by nurses via a face-to-face interview and guided by a standard pro forma paper. Notably, the questions on both the pro forma paper and tablet were identical.

We assessed patient satisfaction with PHA as a quality metric, similar to a previous study [19]. After the nurse–patient consultation, all patients had to complete a survey, consisting of four items measuring general satisfaction, graded on a five-point semantic scale, adopted from a previous questionnaire [22]. We modified one response option from “Definitely” to “Extremely”. For both groups, the duration of the nurse–patient consultation was obtained from the 1 Queue 1 Payment (1Q1P) electronic system. This is an integrated queue and payment system that generates the queue number and electronically measures the timings of all service points for the patient’s appointment, with one consolidated payment at the end. For the study, the duration of the nurse–patient consultation was defined as the time interval from which the nurse first called for the patient to the time at which the patient left the nurse’s station.

### 2.6. Data Analysis

Data were analyzed using IBM SPSS Statistics for Windows, Version 25.0, (IBM corp. Armonk, NY, USA). A descriptive frequency analysis was performed for demographic data. The five-point Likert responses measuring patients’ perceptions of PATCH assessment were further categorized into three categories: (1) a combination of strongly agree and agree, (2) neutral and (3) a combination of disagree and strongly disagree. We felt that this further classification would allow differentiation between favorable (strongly agree and agree) and unfavorable (disagree and strongly disagree) perceptions. Data on the duration of the nurse–patient consultation were examined using the Shapiro–Wilk test for normal distribution. The independent sample *t*-test was used to assess the effect of PATCH on the duration of the nurse consultation.

The median satisfaction score was computed for each patient, as well as the overall satisfaction score for each intervention group. The scores were graded on a five-point scale, ranging from one (“not at all satisfied”) to five (“extremely satisfied”). The minimum possible total score a patient could attain for the four statements was four and the highest was 20. For each statement on satisfaction, we added the scores and then computed the overall median score. For the overall satisfaction score, we added the scores given by each patient to each of the four statements on satisfaction, and computed the median score from the overall satisfaction scores. As the scores were measured on an ordinal scale, the Mann–Whitney test, which computes and compares mean ranks, was applied to evaluate if the differences in patient satisfaction with PHA between groups was significant. The alpha level of significance was set at *p* < 0.05.

## 3. Results

Fifty-two patients were recruited and randomized. Two in the PATCH arm were later excluded: one patient had her surgery changed to inpatient type and the other had completed the pro forma paper for PHA at an earlier visit. Table 1 presents the demographic characteristics of the 50 patients. Patients in the PATCH assessment arm were noted to be younger. More Chinese patients were noted in both arms, compared to other races, and this was consistent with the pattern of ethnic distribution in the country.

The measurement of the duration of the nurse–patient consultation was missed out for one patient in the PATCH arm and two patients in the standard nurse assessment arm. The Shapiro–Wilk test indicated that the duration of nurse consultation time was normally distributed for the PATCH arm (*p* = 0.828) and standard nurse assessment arm (*p* = 0.703). Patients in the PATCH arm had a shorter mean (SD) nurse–patient consultation time of 11.5 (3.6) min, compared to 12.2 (2.9) min for those who underwent standard nurse assessment, but this was not statistically significant (*p* = 0.446).

Results of the perception survey completed by patients in the PATCH arm are shown in Table 2. Overall, favorable perceptions ranged from 41.7% to 79.2%. Seven (29.2%) PATCH users provided suggestions for improving PATCH assessment.

All fifty patients completed a survey evaluating their satisfaction with PHA, as defined by the mode of assessment and quality of the nurse–patient consultation. The median scores pertaining to the four statements were: worries addressed (3.0 vs. 3.5); recommend nurse (4.0 vs. 4.0); satisfied with visit (4.0 vs. 4.0) and feeling prepared (4.0 vs. 4.0). The median overall satisfaction score was slightly lower in the PATCH arm, compared to the standard nurse assessment arm (14.5 vs. 15.5), but this was not statistically significant. Satisfaction scores by item were also comparable between both groups (Table 3).

In the feedback from triage nurses of the PAS, it was observed that nurses felt invested in the project as they recognized its potential in reducing nurses’ burden on PHA, generating time savings and optimizing the utilization of healthcare resources. Two triage nurses also shared their perceptions and provided suggestions to improve PATCH assessment, such as the deletion of the “Unsure” response, the integration of PATCH assessment formally into the patient’s electronic medical records and the inclusion of follow-up questions to qualify and validate responses given as “*Yes*” or “Unsure”. Both nurses acknowledged that PATCH assessment could be completed quicker, compared with standard nurse-led interviews, particularly for patients with no or few co-morbidities.

## 4. Discussion

The results of this study suggest that computerized preanaesthesia health assessment using PATCH can potentially be introduced at our institution, with no difference in nurse–patient consultation times when compared with standard care nurse-led PHA. Patient satisfaction was also comparable between the two modes of screening. A similar conclusion was reached in a previous study that considered the assessment of patient satisfaction as a quality metric [19].

It is somewhat surprising that the present study did not find any significant difference in nurse–patient consultation times between computerized and nurse-led preanaesthesia health assessment. Previous studies that evaluated the effects of tablet- and web-based applications on the duration of PHA had reported a significant reduction of 5 min (*p* < 0.001) and 13 min (*p* < 0.001), respectively, compared to face-to-face assessments [19,23]. We postulate a few reasons for our observations. By virtue of the trial requirements to ensure the safety of patients, we had mandated that nurses validate the responses of patients in the PATCH arm. Hence, during consultation with patients in the PATCH arm, nurses spent time clarifying patient responses and printed out a hard copy of the final response for filing. This verification process could have added time to the consultation and accounted for the lack of difference in nurse–patient consultation times between groups. As the study evolves and the digitized PHA questionnaire becomes validated, the need to clarify and verify responses will diminish, with a consequent shortening of nurse–patient consultation times. The design of our study also differed from those of other studies, in that it was a randomized feasibility trial that employed a standard electronic method for measuring consultation time in real time for both arms. This is in contrast to one study, in which the duration of assessment of standard care patients was not measured in real time, but derived using an equation that adjusted for age and American Society of Anesthesiologists (ASA) status [19]. Hence, findings could have been biased by having mainly ASA classes 1–2 patients in the digital arm, while patients of ASA classes 1–4 received standard care. In another study, consultation times were recorded manually by staff and this could have introduced inaccuracies [23].

The mean duration of healthcare consultations in primary care settings was reported to range from 8.9 to 10 min locally in 2014 [24]. A common limitation faced by public healthcare consultations is time constraints and this could result in patient concerns not being adequately elicited or addressed by the healthcare professional [25]. As it is not known if the change in mode of screening would impact clinic efficiency and throughput, we have sought to compare nurse–patient consultation times as the primary outcome measure. It is, hence, reassuring to note that there was no significant change in consultation times at the preoperative clinic. In fact, it is possible that the quality of nurse–patient consultation could have improved, as the nurses potentially had more time to provide preoperative education. It is likely that the lack of difference in the duration of consultation times could be attributed to nurses apportioning more time to educate PATCH patients on fasting times, perioperative medication intake and discharge planning. In this sense, PATCH self-assessment indirectly confers the benefit of improved focus and quality of nurse consultation. The comparable duration of nurse–patient consultation for both groups supports our hypothesis of equivalence, whereby the response to two treatments is “not too different” or differs only by a clinically unimportant amount [26,27]. One study reported that the time saved could be used efficiently to assess more low-risk patients or focus on higher-risk patients [19].

Our patients’ perceptions of PATCH assessment were generally favorable and similar to those reported in other studies [15,16,18,23]. Although studies may not always indicate the specific aspects of perception assessed, [20] we have sought to measure PATCH’s acceptability by the patients’ willingness to conduct digitized self-assessment in the future and remotely. Seventy-nine percent of patients in the PATCH assessment arm were willing to use PATCH in the future. This compares favorably with the positive response rates of 59% to 98% reported in previous studies [15,16,18]. About 62% of study patients were willing to complete the assessment remotely and this, too, resonated with a similar study in which more than 50% showed interest in completing the assessment remotely before the clinic visit [23]. Patients’ acceptance of digitized methods of health assessment could spur the development of care pathways that would enhance patient convenience and improve healthcare efficiency. For instance, patients listed for surgery could complete an online PHA process remotely and only those identified to have medical issues need to be assessed in an outpatient setting. Medically stable or healthy patients are allowed to bypass outpatient assessment and present themselves on the day of surgery. Such a new care pathway could save patients an unnecessary hospital visit and allow healthcare resources to be utilized more efficiently.

Preanaesthesia health assessment could be further complemented by other digital modalities, such as email, telephone and video consultations, to follow up on abnormal responses and address patients’ concerns. This may prove viable locally, as statistics indicate that Singaporeans have an average of 3.3 devices per person, with smartphones being the most common (85%), followed by personal computers (74%) and tablets (40%) [28]. Moreover, information collected via digital means might be more complete and reliable, compared to that obtained through a face-to-face interview, as patients are more willing to share their health information digitally [25,29,30]. This was verified in our study, in which 58.3% of patients in the PATCH group reported to have provided greater detail of their medical history, compared to traditionally conducted consultations. In addition, digital self-assessment could lead to improved quality of healthcare consultation, as the questions provide scope and focus to guide the consultation [25,29]. We had sought to explore if PATCH could deliver on these expectations and found that more than half (54.2%) of patients in the PATCH arm reported being able to prepare questions and identify concerns or topics in advance of their consultation with the nurse.

Of note, only 42% of patients agreed or strongly agreed that they felt less anxious after using PATCH, while 54% were neutral. It is widely recognized that patients may feel anxious before elective surgery and a reduction in pre-operative anxiety levels may be associated with better outcomes [22,31,32]. As patients at our hospital are routinely provided with information pamphlets about their upcoming surgery, we speculate that this move could have helped to allay their anxiety, as supported by studies [33].

Feedback from nurses to integrate PATCH assessments into the patients’ electronic health records was a valid and important one, as it ensures a seamless flow of health information to facilitate efficient diagnosis and management. Although the National Electronic Health Records (NEHR) in Singapore does not currently allow patients to contribute directly to their electronic health records, patients have contributed to their electronic health records in other settings [34]. A recent study in patients with psoriasis reported a high acceptance rate (84%) for patients to enter data directly into an EHR and from home (72.2%) [35]. The study team is currently working with the hospital’s medical informatics department to explore this initiative.

The digital acquisition of self-reported patient data has been implemented in primary care, emergency departments and sexual health clinics [29,36,37,38,39,40,41], where it has been shown to improve efficiency in assessing and screening patients’ health. Future research should seek to further validate the use of digital health assessment, with considerations for socio-cultural and economic factors in its implementation. By incorporating a visual analogue scale (VAS) and information aimed at reducing patient anxiety levels, PATCH could be extended to manage preoperative patient anxiety and improve patients’ knowledge on the perioperative process. Alternatively, other modes of telehealth or eHealth communication (e.g., chatbots) may be integrated into PATCH for patient education. Beyond its utility as a digital tool for data acquisition, PATCH could be developed into a digital health assistant, capable of performing thorough assessment and preoperative risk prediction by the incorporation of a clinical decision support system (CDSS), e.g., in predicting ASA physical classification and obstructive sleep apnoea. The study team plans to further validate patient responses to digitized questions and identify those that require “branching” questions to probe “Yes” and “Unsure” responses. This would allow the gathering of more in-depth information, pertinent to the perioperative period. Further research should also determine if digitized PHA improves the cost effectiveness of perioperative processes, such as a reduction in surgical case cancellations.

There are limitations to this study. As we have sampled only adult female patients attending the PAS in one hospital, the findings cannot be generalized to other hospitals or male patients. As 83% of the resident population in Singapore was reported to be literate in English, we recruited only patients literate in English in this pilot study [42]. The findings could be different if the questions were translated and extended to patients proficient in other languages. The small pilot sample size did not permit sub-group analysis and multivariate analysis could not be performed. Follow-up studies would be needed to validate the results.

## 5. Conclusions

Compared to standard care nurse-led preanaesthesia health assessment, patient self-assessment using a digitized application can feasibly be introduced into current practice with a comparable duration of nurse–patient consultation and patient satisfaction.

## Figures and Tables

**Table 1:** Demographic characteristics at study entry (*n* = 50).

	PATCH Assessment (*n* = 24)	Standard Nurse Assessment (*n* = 26)
Age, years (mean {SD})	35.5 (8.3)	41 (11)
Educational level		
Primary to post-secondary	7 (29.2%)	17 (65.4%)
Polytechnic to post-university	17 (70.8%)	9 (36%)
Ethnicity		
Chinese	15 (62.5%)	11 (42.3%)
Other ethnicities	9 (37.5%)	15 (57.7%)

PATCH: PreAnaesThesia Computerized Health; SD: standard deviation.

**Table 2:** Patients’ perceptions of PATCH assessment (*n* = 24).

	Strongly Agree—Agree *n* (%)	Neutral *n* (%)	Strongly Disagree—Disagree *n* (%)
I would use PATCH again in the future.	19 (79.2%)	5 (20.8%)	0 (0%)
I would like to use PATCH at home or from work before coming to clinic.	16 (66.7%)	7 (29.2%)	1 (4.1%)
I was able to provide a greater level of detail (tell my history better) compared to past consultations I had with a doctor or nurse.	14 (58.4%)	7 (29.2%)	3 (12.5%)
I was able to prepare questions, identify concerns and/or topics about my medical history to raise and discuss with the nurse.	13 (54.1%)	11 (45.8%)	0 (0%)
I was able to organize my thoughts before meeting the nurse.	13 (54.1%)	11 (45.8%)	0 (0%)
Compared to past medical interviews, I liked using PATCH to describe my medical history without undue influence from the nurse.	13 (54.1%)	10 (41.7%)	1 (4.2%)
It helped me to think of questions or concerns that I hadn’t thought of before.	12 (50%)	10 (41.7%)	2 (8.3%)
I felt less anxiety after using PATCH.	10 (41.7%)	13 (54.2%)	1 (4.2%)

**Table 3:** Median satisfaction scores in PATCH and standard nurse assessment groups.

	PATCH Assessment (*n* = 24)	Standard Nurse Assessment (*n* = 26)	*p*-Value
My worries about the operation have been addressed today.	23.4	27.5	0.304
I would recommend my nurse.	23.9	27.0	0.437
I am satisfied with my visit to the clinic.	24.8	26.2	0.716
I am prepared for my anaesthetic/operation.	24.3	26.6	0.559
Overall satisfaction	23.9	27.0	0.451
